# Phenotyping physician practice patterns and associations with response to a nudge in the electronic health record for influenza vaccination: A quasi-experimental study

**DOI:** 10.1371/journal.pone.0232895

**Published:** 2020-05-20

**Authors:** Sujatha Changolkar, Jeffrey Rewley, Mohan Balachandran, Charles A. L. Rareshide, Christopher K. Snider, Susan C. Day, Mitesh S. Patel

**Affiliations:** 1 Penn Medicine Nudge Unit, University of Pennsylvania, Philadelphia, Pennsylvania, United States of America; 2 Crescenz Veterans Affairs Medical Center, Philadelphia, Pennsylvania, United States of America; 3 Perelman School of Medicine, University of Pennsylvania, Philadelphia, Pennsylvania, United States of America; 4 Wharton School, University of Pennsylvania, Philadelphia, Pennsylvania, United States of America; University of New South Wales, AUSTRALIA

## Abstract

**Background:**

Health systems routinely implement changes to the design of electronic health records (EHRs). Physician behavior may vary in response and methods to identify this variation could help to inform future interventions. The objective of this study was to phenotype primary care physician practice patterns and evaluate associations with response to an EHR nudge for influenza vaccination.

**Methods and findings:**

During the 2016–2017 influenza season, 3 primary care practices at Penn Medicine implemented an active choice intervention in the EHR that prompted medical assistants to template influenza vaccination orders for physicians to review during the visit. We used latent class analysis to identify physician phenotypes based on 9 demographic, training, and practice pattern variables, which were obtained from the EHR and publicly available sources. A quasi-experimental approach was used to evaluate response to the intervention relative to control practices over time in each of the physician phenotype groups. For each physician latent class, a generalized linear model with logit link was fit to the binary outcome of influenza vaccination at the patient visit level. The sample comprised 45,410 patients with a mean (SD) age of 58.7 (16.3) years, 67.1% were white, and 22.1% were black. The sample comprised 56 physicians with mean (SD) of 24.6 (10.2) years of experience and 53.6% were male. The model segmented physicians into groups that had higher (*n* = 41) and lower (*n* = 15) clinical workloads. Physicians in the higher clinical workload group had a mean (SD) of 818.8 (429.1) patient encounters, 11.6 (4.7) patient appointments per day, and 4.0 (1.1) days per week in clinic. Physicians in the lower clinical workload group had a mean (SD) of 343.7 (129.0) patient encounters, 8.0 (2.8) patient appointments per day, and 3.1 (1.2) days per week in clinic. Among the higher clinical workload group, the EHR nudge was associated with a significant increase in influenza vaccination (adjusted difference-in-difference in percentage points, 7.9; 95% CI, 0.4–9.0; *P* = .01). Among the lower clinical workload group, the EHR nudge was not associated with a significant difference in influenza vaccination rates (adjusted difference-in-difference in percentage points, -1.0; 95% CI, -5.3–5.8; *P* = .90).

**Conclusions:**

A model-based approach categorized physician practice patterns into higher and lower clinical workload groups. The higher clinical workload group was associated with a significant response to an EHR nudge for influenza vaccination.

## Introduction

Nearly 90% of primary care physicians (PCPs) in the United States use an electronic health record (EHR) to facilitate medical decision-making [1,2]. Health systems are increasingly implementing changes to the design of EHRs to influence physician behavior [3]. These interventions are typically deployed broadly to all physicians within a clinical specialty or throughout the entire health system [4]. In some cases these may lead to benefits for the overall group. However, there may be some physicians for whom these interventions are not effective. Moreover, for some physicians, these design changes could have a negative impact either directly on the targeted behavior or indirectly on other behaviors. However, there is a lack of evidence on methods to identify groups of physicians with differential responses to these types of interventions.

Existing data from EHRs could be used to identify physicians with different behavioral phenotypes. For example, physician practices patterns may vary in terms of the volume or types of patients they provide care for in clinic. Model-based approaches that could segment physicians into different phenotype groups may allow for tailoring of behavioral interventions to improve patient care. For example, latent class analysis has been used to classify phenotypes using clinical [5,6], behavioral [7,8], and activity data [9,10].

Nudges are subtle changes to the design of choice architecture that can have a significant impact on behavior [3]. In prior work by members of our group, we found that an active choice intervention in the EHR to prompt medical assistants to template influenza vaccination orders for physicians during primary care visits led to a 9.5-percentage point increase in influenza vaccination in intervention practices relative to control practices over time [11]. However, physicians may have varied in their response to this intervention. In this study, our objective was to phenotype physicians using EHR data on their practice patterns and then evaluate associations with responses to an active choice nudge in the EHR for influenza vaccination.

## Methods

The University of Pennsylvania Institutional Review Board approved this study and waived informed consent because it was infeasible given the study design, and the study posed minimal risk.

### Setting and participants

Similar to prior work [11], the sample comprised primary care physicians (PCPs) from 10 primary care practices (3 intervention, 7 control) at Penn Medicine and patients who visited those PCPs during two influenza seasons (September 1^st^ to March 31^st^) between 2015 and 2017. We excluded PCPs who did not see patients during the entire practice period or had at least one month during the 2015–16 influenza season (pre-intervention period) without any patient visits (*n* = 7). We evaluated each patient’s first new or return visit with their PCP during the study period. Acute, sick, or other visits were excluded because influenza vaccination may not be appropriate at those times. Patients were excluded if EHR documentation indicated they were already vaccinated prior to the visit.

### Intervention

Prior to the intervention, PCPs had to remember to manually check if a patient was due for influenza vaccination, discuss it with the patient, and then place an order for it in the EHR. During the 2016 to 2017 influenza season, three Penn Medicine primary care practices implemented an active choice intervention in the EHR using a best practice alert in Epic, directed to medical assistants [11]. Prior to meeting with the physician, patients met with a medical assistant to check their vitals. At that time, the EHR assessed patient eligibility for the influenza vaccine and prompted medical assistants to accept or cancel an order for the vaccine. If accepted, the order was templated for the physician to review and sign during the patient visit. The control group comprised seven primary care clinics that did not implement the intervention.

### Data

Clarity, an Epic reporting database, was used to obtain data on physicians (demographics, training, and practice patterns), patients (demographics, insurance, comorbidities, prior influenza vaccination status, and PCP), and clinic visits (date, appointment time, practice site, visit type, and presence of an order for influenza vaccination or not). Publicly available data sources were used for information on physician years of experience and sex. U.S. Census data was used to find the median household income by zip code, when available.

Physician practice attributes were defined as follows. Physician years of experience was calculated as the number of years a physician had been in practice between earning a medical degree and the beginning of the study period (2015). The number of encounters was the total number of patient visits for a physician at Penn Medicine during the 2015–2016 influenza season. For each physician, we estimated the mean Charlson Comorbidity Index [12] (CCI) among their patients. Each physician’s average number of appointments per hour was estimated by first identifying the individual hours of day they worked during the influenza season. Any hour of the day that amounted to fewer than 5% of total hours (typically hours like 6:00 am) were dropped. Finally, the physician’s total number of completed appointments was divided by the distinct hours in which they saw patients. We estimated the mean number of days per week a physician saw patients in clinic. Delay was estimated as the mean number of minutes between the scheduled appointment and physician opening the patient chart; we excluded outliers attributed to miscoding of data in the EHR [13]. The percent of new patient encounters for each physician was estimated using visit type data. To capture differences in vaccination rates by appointment time [11,14], the percent of visits in the morning (after 8am and before 1pm) was calculated out of the total number of visits between 8am and 6pm for each physician.

### Statistical analysis

The primary outcome measure was the presence of an order for influenza vaccination. In prior work, over 99.9% of vaccine orders also had an insurance claim indicating the vast majority of these orders resulted in actual vaccination [15]. Insurance information was not available for this study.

To phenotype physician practice patterns, we used latent class analysis (LCA) which is a model-based approach that uses observable variables to classify individuals into previously unmeasured subgroups [16]. Variables are identified as latent class indicators with the goal of distinguishing between classes and categorizing physicians into their most likely classes given observed data [17]. The following nine physician variables were used in the LCA: years of experience, sex, number of patient encounters, mean CCI of patient encounters, mean number of patient appointments per weekday, mean number of weekdays in clinic per week, mean minutes of appointment delay, percent of new patient visits, and percent of visits per weekday in the morning before 1pm. Variable distributions were assessed to inform balanced categorization of continuous variables.

To identify the optimal number of latent classes, we used several measures to assess model fit [18]. The Bayesian information criterion (BIC) was used to evaluate goodness of model fit [19]. The parametric bootstrapped likelihood ratio test (LRT) was used to assess whether a given model with *k* classes is significantly more informative than one with *k*-1 classes [20]. Entropy was used to evaluate distinctness between classes [21]. We also required that each class have at least 5% of physicians to prevent underrepresentation of certain characteristics. LCA modeling was conducting using MPlus (Version 8.2) [21].

To evaluate the association of physician phenotype classification with response to the intervention, we used a difference-in-differences approach [22], as used in prior work [11,15,23]. Changes in influenza vaccination between groups (intervention versus control practices) and time (post-intervention year versus pre-intervention year) were compared for each latent class. A generalized linear model with logit link was fit to the binary outcome of influenza vaccination at the patient visit level for each class of physicians. These models were adjusted for patient demographics (age, sex, race/ethnicity), CCI [12], and insurance type. The models were adjusted by practice site and month fixed effects, an interaction term for year and group, and were clustered by physician. The adjusted difference-in-difference in percentage points with 95% confidence intervals were generated using the bootstrapping procedure [24,25], resampling patients 1000 times. Resampling of patients was conducted by physician to maintain clustering at the physician level. Two-sided hypothesis tests used a significance level of 0.05. Regression analyses were conducted in R (Version 3.5.1; R Foundation for Statistical Computing).

## Results

### Sample description

The sample comprised 56 physicians with mean (SD) of 24.6 (10.2) years of experience and 53.6% were male (Table 1). These physicians had a mean (SD) number of 819 (429) patient encounters, 11.6 (4.7) appointments per day, and 4.0 (1.1) days per week in clinic. The sample comprised 45,410 patients with mean (SD) age of 58.7 (16.3) years, 67.1% were white, and 22.1% were black (Table 2).

**Table 1:** Sample characteristics for physicians within identified practice pattern phenotype groups.

Characteristic	Lower Clinical Workload Physician Group (Class 1)	Higher Clinical Workload Physician Group (Class 2)	*P* Value	All Physicians
Physicians, *n* (%)	15 (100.0)	41 (100.0)	NA	56 (100.0)
Intervention Practices	12 (80.0)	18 (43.9)		30 (53.6)
Control Practices	3 (20.0)	23 (56.1)		26 (46.4)
Years of experience, mean (SD)	24.0 (10.4)	24.8 (10.2)	0.81	24.6 (10.2)
Male gender, *n* (%)	8 (53.3)	22 (53.7)	1.00	30 (53.6)
Encounters, mean (SD)	343.7 (129.0)	992.7 (362.9)	<.001	818.8 (429.1)
Appointments per day, mean (SD)	8.0 (2.8)	12.9 (4.6)	<.001	11.59 (4.7)
Days in clinic, mean (SD)	3.1 (1.2)	4.3 (0.8)	<.001	4.0 (1.1)
Delay in minutes, mean (SD)	33.9 (16.6)	26.4 (16.8)	0.16	28.4 (16.9)
New patients, %	4.1	9.4	<.001	8.8
Morning visits 8am to 12pm, %	63.8	60.3	<.001	60.7
Encounters Charlson Comorbidity Index, median (IQR)	1.9 (0.3)	1.5 (0.8)	0.005	1.7 (0.6)

*Abbreviations: SD = standard deviation; IQR = interquartile range; NA = not applicable.

**P Value compares Lower Clinical Workload Physician Group to Higher Clinical Workload Physician Group.

**Table 2:** Sample characteristics for patients.

	Lower Clinical Workload Physician Group		Higher Clinical Workload Physician Group		Total, All Years
	2015–2016 (Pre)	2016–2017 (Post)	2015–2016 (Pre)	2016–2017 (Post)	
New/Return Visits, *n* (%)	2532 (100.0)	2315 (100.0)	20672 (100.0)	19891 (100.0)	45410 (100.0)
Intervention practices	1974 (78.0)	1799 (77.7)	6397 (30.9)	5342 (26.9)	15512 (34.2)
Control practices	558 (22.0)	516 (22.3)	14275 (69.1)	14549 (73.1)	29898 (65.8)
Age, mean (SD)	60.1 (15.3)	60.9 (14.8)	58.1 (16.5)	59.0 (16.3)	58.7 (16.3)
Female gender, *n* (%)	1546 (61.1)	1420 (61.3)	11405 (55.2)	10784 (54.2)	25155 (55.4)
Race/ethnicity, *n* (%)					
White non-Hispanic	1331 (52.6)	1206 (52.1)	14256 (69.0)	13683 (68.8)	30476 (67.1)
Black non-Hispanic	952 (37.6)	875 (37.8)	4146 (20.1)	4042 (20.3)	10015 (22.1)
Asian	118 (4.7)	98 (4.2)	542 (2.6)	521 (2.6)	1279 (2.8)
Hispanic	26 (1.0)	27 (1.2)	250 (1.2)	219 (1.1)	522 (1.1)
Other	105 (4.1)	109 (4.7)	1478 (7.1)	1426 (7.2)	3118 (6.9)
Insurance, *n* (%)					
Commercial	1370 (54.1)	1195 (51.6)	12660 (61.2)	11767 (59.2)	26992 (59.4)
Medicare	996 (39.3)	974 (42.1)	7297 (35.3)	7431 (37.4)	16698 (36.8)
Medicaid	166 (6.6)	146 (6.3)	715 (3.5)	693 (3.5)	1720 (3.8)
Annual household income, mean (SD)	62708 (35188)	77177 (32199)	63008 (34675)	76849 (31934)	75503 (32682)
Charlson Comorbidity Index, median (IQR)	1 (3)	1 (4)	1 (2)	1 (2)	1 (2)

*Abbreviations: SD = standard deviation; IQR = interquartile range.

### Identifying phenotypes

The two-class model had good fit with a BIC of 906.1, an entropy of 1.0, and a significant parametric bootstrapped likelihood ratio test (*P*<.001) when compared to 3- and 4-class models (S1 Table). Class 1 comprised 15 physicians with a mean (SD) of 343.7 (129.0) patient encounters, 8.0 (2.8) patient appointments per day, and 3.1 (1.2) days per week in clinic. Class 2 comprised 41 physicians with a mean (SD) of 818.8 (429.1) patient encounters, 11.6 (4.7) patient appointments per day, 4.0 (1.1) days per week in clinic. These classes varied in their level of workload and therefore were labeled as lower clinical workload (Class 1) and higher clinical workload (Class 2) (Table 1). Among the 15 physicians in the lower clinical workload group, 3 were in the intervention practices and 12 were in control practices. Among the 41 physicians in the higher clinical workload group, 23 were in the intervention practices and 18 were in control practices.

### Physician phenotypes and changes in influenza vaccination

**Fig 1:**
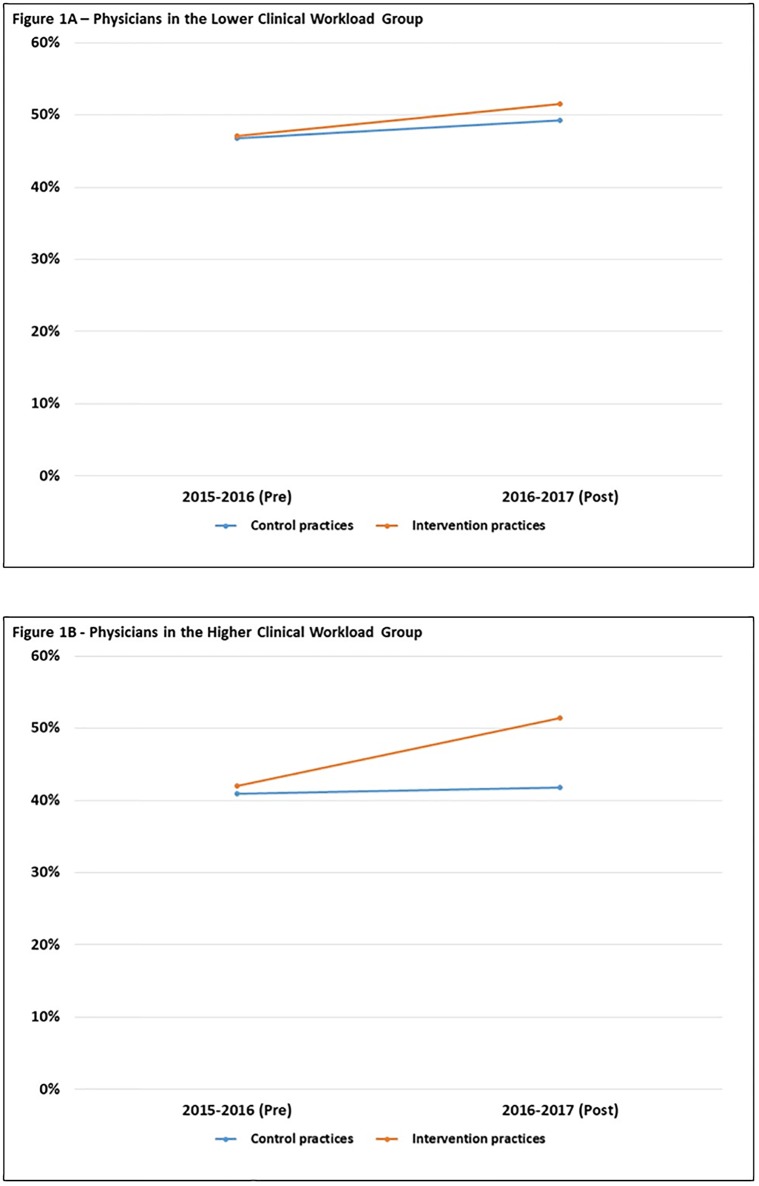
Influenza vaccination rates by practice group and year. The unadjusted percentage of patients that received influenza vaccination among physicians in the lower clinical workload group (A) and physicians in the higher clinical workload group (B). The active choice intervention was implemented at the intervention practices during the 2016–2017 year.

Influenza vaccination rates for the lower clinical workload group at control practices were 47.8% in 2015–16 and 49.2% in 2016–17, and at intervention practices were 47.1% in 2015–16 and 51.5% in 2016–17. For the higher clinical workload group, influenza vaccination rates at control sites were 40.9% in 2015–16 and 41.8% in 2016–17, and at intervention practices were 42.0% in 2015–16 and 51.4% in 2016–17. The unadjusted difference for intervention versus control sites in the intervention period relative to the pre-intervention period was 8.6-percentage points for higher clinical workload group and 1.9-percerntage points for the lower clinical workload group (Fig 1).

Among the higher clinical workload group, the EHR nudge was associated with a significant increase in influenza vaccination (adjusted difference-in-difference in percentage points, 7.9; 95% CI, 0.4–9.0; *P* = .01) (Table 3). Among the lower clinical workload group, the EHR nudge was not associated with a significant difference in influenza vaccination rates (adjusted difference-in-difference in percentage points, -1.0; 95% CI, -5.3–5.8; *P* = .90). Regression tables for both difference-in-difference models are available (S2 and S3 Tables).

**Table 3:** Adjusted difference in influenza vaccination among physician practice pattern phenotype groups.

	Adjusted Difference-in-Difference (95% CI), Percentage Points	*P* Value
Lower Clinical Workload Physician Group	-1.0 (-5.3, 5.8)	.90
Higher Clinical Workload Physician Group	7.9 (0.4, 9.0)	.01

## Discussion

In this study of 10 primary care practices, we found that a model-based approach categorized physician practice patterns into higher and lower clinical workload groups. While a prior study among these practices found that an EHR-based nudge increased influenza vaccination rates relative to control practices over time, we found differential responses based on the identified physician subgroups. The intervention was associated with a significant increase in influenza vaccination among physicians in the higher clinical workload group, but not among those in the lower workload group. To our knowledge, this is one of first studies use this type of approach to phenotype physician practice patterns and compare responses to a behavioral intervention.

These findings have several important implications. First, behavioral phenotyping has been described previously, but mostly in the context of identifying patients with differential response to failure [26,27]. In this study, we used available EHR data to identify physicians with different practice pattern phenotypes. Since more than 90% of health systems use EHRs, this is a scalable approach that could be applied to other areas of health care.

Second, the design of the nudge intervention may reveal insights into mechanisms for differential responses between the physician groups. Most patients that present to primary care visits during influenza season are eligible for vaccination if they have not already received it. The EHR intervention was delivered to medical assistants who could template vaccination orders for physicians to review and discuss with patients [11]. Among physicians with higher clinical workloads, this may have helped alleviate the effort needed to do this consistently for patients throughout the day. Physicians with higher patient volumes may be more likely than physicians with lower clinical workloads to face decision fatigue, which is the depletion of self-control and active initiative that results from the cumulative burden of making decisions [28]. They may also be more likely to fall behind schedule as the day progresses. Our prior work has shown that these two factors can lead to lower vaccination rates and well as worsening in other aspects of care such as cancer screening [11,14].

Third, we identified that about 27% of physicians in the overall sample had no benefit from the intervention. While the difference between intervention and control was not significant for the lower clinical workload group, these physicians started at a higher baseline vaccination rate, which the intervention helped higher workload clinicians reach, but not exceed. EHR-based interventions have been known to create alert fatigue and this could be an opportunity to reduce this burden among these physicians and their staff [29–31]. It could also allow for the recognition that another form of intervention may be better suited to nudge physicians with this lower clinical workload phenotype to improve vaccination rates. Additionally, influenza vaccination rates might be further enhanced if coupled with a patient-facing nudge.

This study has limitations. First, any observational study is susceptible to unmeasured confounders. However, we used a difference-in-differences approach which reduces potential bias from unmeasured confounders by comparing changes in vaccination over time between intervention and control practices. Second, this study was conducted within a single health system, which may limit generalizability. However, we included 10 practice sites from 2 different states. Third, we evaluated influenza vaccination order status at the time of first visit during influenza season, but patients who subsequently receive a timely influenza vaccination were not captured in this study. Fourth, while we were able to identify physician subgroups with differential response to the intervention, our study design did not evaluate specific mechanisms that led to these responses.

## Conclusions

A model-based approach categorized physician practice patterns into higher and lower clinical workload groups. The EHR nudge was associated with a significant increase in influenza vaccination orders among physicians in the higher clinical workload group, but not among those in the lower workload group. This approach could be used in other areas of health care to identify variation in response and better design the targeting of future interventions.

## Supporting information

S1 Table Model fit criteria. (XLSX)

S2 Table Regression lower clinical workload. (XLSX)

S3 Table Regression higher clinical workload. (XLSX)

S4 Table Variable weights by class. (XLSX)
